# Supplementary material for: Virus infections in honeybee colonies naturally surviving ectoparasitic mite vectors
Source: PLoS One. 2023 Dec 15;18(12):e0289883. doi: 10.1371/journal.pone.0289883 (PMC10723705; doi:10.1371/journal.pone.0289883)
Supplement: S1 Table — Parameters include: the population type (treated or untreated), “Season” is the time of year the samples were taken (Spring, Summer or Autumn), “Species”, whether the organism screened was a honeybee or a V. destructor mite and “Brood/Worker infestation” The V. destructor infestation rate sampled per colony in either brood or adult worker bees. (PDF) [file pone.0289883.s001.pdf]

S1 Table: Model output for the full analysis. Parameters include: the population type (treated or untreated), "Season" is the time of year the samples were taken (Spring, Summer or Autumn), "Species", whether the organism screened was a honeybee or a *V. destructor* mite and "Brood/Worker infestation" The *V. destructor* infestation rate sampled per colony in either brood or adult worker bees.

Worker infestation rate					
Model	Dependent	Independent	df	test stat (F)	p value
M1a	Worker infestation	Population type:Season	1	1.9	0.17
		Population type	1	5.88	0.017*
		Season	2	3.38	0.068

Brood infestation rate					
Model	Dependent	Independent	df	t-value	p value
M2a	Brood infestation	Population type	1	1.18	0.073
		Season	2	2.24	0.017*

Pooled adult worker and mite prevalence BQCV (from qpcr negative results)					
Model	Dependent	Independent	df	test stat (χ^2)	p value
M3a	BQCV presence	Species	1	49.18	<0.001***
		Population type	1	15.03	<0.001***

Pooled adult worker and mite prevalence DWVA (from qpcr negative results)					
Model	Dependent	Independent	df	test stat (χ^2)	p value
M3b	DWV-A presence	Species	1	0.009	0.92
		Population type	1	4.61	0.03*

Pooled adult worker and mite prevalence LSV1 (from qpcr negative results)					
Model	Dependent	Independent	df	test stat (χ^2)	p value
M3c	LSV1 presence	Species	1	8.7	0.003**
		Population type	1	1.19	0.28

Pooled adult worker and mite prevalence LSV2 (from qpcr negative results)					
Model	Dependent	Independent	df	test stat (χ^2)	p value
M3d	LSV2 presence	Species	1	68.87	<0.001***
		Population type	1	2.66	0.1

Pooled adult worker and mite prevalence SBPV (from qpcr negative results)

Model	Dependent	Independent	df	test stat (χ^2)	p value
M3e	SBPV presence	Species	1	16.09	<0.001***
		Population type	1	2.39	0.12

Pooled adult worker viral abundance					
Model	Dependent	Independent	df	test stat (F)	p value
M4a	Viral abundance	Brood infestation:Worker infestation	1	3.95	0.049
		Brood infestation	1	7.54	0.007**
		Worker infestation	1	2.25	0.14
		Virus: Population type	4	4.91	0.001**

Pooled adult worker post-hoc abundance analysis BQCV					
Model	Dependent	Independent	df	test stat (F)	p value
M4b	Viral abundance	Brood infestation	1	0.16	0.69
		Population type	1	39.64	<0.001***

Pooled adult worker post-hoc abundance analysis DWV-A					
Model	Dependent	Independent	df	test stat (F)	p value
M4c	Viral abundance	Brood infestation	1	8.18	0.01*
		Population type	1	0.41	0.53

Pooled adult worker post-hoc abundance analysis LSV1					
Model	Dependent	Independent	df	test stat (F)	p value
M4d	Viral abundance	Brood infestation	1	55.56	<0.001***
		Population type	1	0.44	0.53

Pooled adult worker post-hoc abundance analysis LSV2					
Model	Dependent	Independent	df	test stat (F)	p value
M4e	Viral abundance	Brood infestation	1	0.2	0.66
		Population type	1	2.3	0.14

Pooled adult worker post-hoc abundance analysis SBPV					
Model	Dependent	Independent	df	test stat (F)	p value

M4f	Viral abundance	Brood infestation	1	5.53	0.038
		Population type	1	5.8	0.035

Individual worker brood and mite prevalence DWV-A					
Model	Dependent	Independent	df	test stat (χ^2)	p value
M5a	DWV-A presence	Population type	1	6.75	0.009**
		Season	2	28.64	<0.001***
		Pupa age	1	2.84	0.09
		Species	1	8.7	0.003**

Individual worker brood and mite prevalence DWV-B					
Model	Dependent	Independent	df	test stat (χ^2)	p value
M5b	DWV-B presence	Population type	1	0.21	0.65
		Season	2	5.63	0.06
		Pupa age	1	13.41	<0.001***
		Species	1	2.95	0.086

Individual worker brood and mite prevalence SBPV					
Model	Dependent	Independent	df	test stat (χ^2)	p value
M5c	SBPV presence	Population type	1	1.8	0.18
		Season	2	16.44	<0.001***
		Pupa age	1	1.12	0.29
		Species	1	0.18	0.67

Individual viral abundance pupae and mites DWV-A					
Model	Dependent	Independent	df	test stat (F)	p value
M6a	Viral abundance	Mite rep. status	1	0.19	0.67
		Age of pupa	1	13.2	<0.001***
		Species	1	9.53	0.002**
		Season: Population type	1	2.1	0.65
		Season	2	12.57	<0.001***
		Population type	1	19.78	<0.001***

Individual viral abundance pupae and mites DWV-B					
Model	Dependent	Independent	df	test stat (F)	p value
M6b	Viral abundance	Mite rep. status	1	0.55	0.46
		Age of pupa	1	0.54	0.46

		Species	1	5.16	0.024*
		Season: Population type	1	0.52	0.47
		Season	2	11.48	<0.001***
		Population type	1	0.6	0.44

Individual viral abundance pupae and mites SBPV					
Model	Dependent	Independent	df	test stat (F)	p value
M6c	Viral abundance	Mite rep status	1	1.07	0.3
		Age of pupa	1	1.5	0.22
		Species	1	101.93	<0.001***
		Season: Population type	1	1.28	0.26
		Season	2	36.23	<0.001***
		Population type	1	1.91	0.7

Individual viral abundance MITES ONLY DWV-A					
Model	Dependent	Independent	df	test stat (F)	p value
M6d	Viral abundance	Mite rep status	1	0.95	0.33
		Age of pupa	1	8.73	0.004**
		Season: Population type	1	0.03	0.86
		Season	2	43.66	<0.001***
		Population type	1	11.13	0.001**
