# Supplementary material for: Virus infections in honeybee colonies naturally surviving ectoparasitic mite vectors
Source: PLoS One. 2023 Dec 15;18(12):e0289883. doi: 10.1371/journal.pone.0289883 (PMC10723705; doi:10.1371/journal.pone.0289883)
Supplement: S2 Table — (PDF) [file pone.0289883.s002.pdf]

S2 Table. Primers used for the qualitative and quantitative detection of bee viruses.

	Target	Primer name	Sequence (5'-3')	Product size (bp)	Ref.
Qualitative PCR assays	DWV-A	DWV-F1425<br>DWV-B1806	CGTCGGCCTATCAAAG<br>CTTTTCTAATTCAACTTCACC	417	(de Miranda & Fries, 2008)
	DWV-B	VDV1-F1409<br>DWV-B1806	GCCCTGTTCAAGAACATG<br>CTTTTCTAATTCAACTTCACC	412	(de Miranda & Fries, 2008)
	ABPV	ABPF-F<br>ABPV-R	CTCAAGTTATACGTAAAATAGCTGGA ATT<br>AACCAACCTTGCTTCCCTTTA	646	(Tentcheva *et al.*, 2004)
	IAPV	IAPV-F6627<br>IAPV-R6707	CCATGCCTGGCGATTCAC<br>CTGAATAATACTGTGCGTATC	203	(Gauthier *et al.*, 2011)
	KBV	KBV-F<br>KBV-R	GATGAACGTCGACCTATTGA<br>CTGAATAATACTGTGCGTATC	414	(Tentcheva *et al.*, 2004)
	CBPV	CBPV-F<br>CBPV-R	AGTTGTCATGGTTAACAGGATACGAG<br>TCTAATCTTAGCACGAAAGCCGAG	455	(Tentcheva *et al.*, 2004)
	SBV	SBV-F<br>SBV-R	GGATGAAAGGAAATTACCAG<br>CCACTAGGTGATCCACACT	426	(Tentcheva *et al.*, 2004)
	BQCV	BQCV-F<br>BQCV-R	GTCCAGTGTGATATTGCCAA<br>TCATTAGAAAGCGCCAGACT	550	(Tentcheva *et al.*, 2004)
	SBPV	SBPV-F3177<br>SBPV-B3363	GCGCTTTAGTTCAATTGCC<br>ATTATAGGACGTGAAAATATAC	226	(de Miranda *et al.*, 2010)
	LSV 1	qLSV1-F2569<br>qLSV1-R2743	AGAGGTTGCACGGCAGCATG<br>GGGACGCAGCACGATGCTCA	174	(Runckel *et al.*, 2011)
	LSV 2	LSV2-F3954<br>LSV2-R4512	CGGCCGGTCTAGCGTGGTTG<br>TGGCAAGCTGTGACGAATCCCT	558	(Runckel *et al.*, 2011)
Quantitative PCR assays	DWV-A	DWVQ_F1<br>DWVQ_R1	TAGTGCTGGTTTTCCTTTGTC<br>CTGTGTCGTTGATAATTGAATCTC	145	(Highfield *et al.*, 2009)
	DWV-B	VDV-1 NS F<br>VDV-1 NS R	TTCATTAAAACCGCCAGGCTCT<br>CAAGTTCAGGTCTCATCCCTCT	100	(Moore *et al.*, 2011)
	BQCV	BQCV-qF7893<br>BQCV-qB8150	AGTGGCGGAGATGTATGC<br>GGAGGTGAAGTGGCTATATC	294	(Locke *et al.*, 2012)
	SBPV	SBPV-F3177<br>SBPV-B3363	GCGCTTTAGTTCAATTGCC<br>ATTATAGGACGTGAAAATATAC	226	(de Miranda *et al.*, 2010)
	LSV 1	qLSV1-F2569<br>qLSV1-R2743	AGAGGTTGCACGGCAGCATG<br>GGGACGCAGCACGATGCTCA	174	(Runckel *et al.*, 2011)
	LSV 2	qLSV2-F1722<br>qLSV2-R1947	CGTGCTGAGGCCACGGTTGT<br>GCGGTGTCGATCTCGCGGAC	226	(Runckel *et al.*, 2011)
	β-Actin (*A.m.*)	*A.m.* β-Actin-q92F<br>*A.m.* β-Actin-q157R	CGTTGTCCCGAGGCTCTTT<br>TGTCTCATGAATACCGCAAGCT	66	(Gauthier *et al.*, 2011)
	β-Actin (*V.d.)*	Vd-actin-qF<br>Vd-actin-qB	CGACGGTCAGGTCATCAC<br>GTTGAGGGAGCCAAAGAGG	243	(Locke *et al.*, 2012)

De Miranda, J.R., Dainat, B., Locke, B., Cordoni, G., Berthoud, H., Gauthier, L., et al. 2010. Genetic characterization of slow bee paralysis virus of the honeybee (Apis mellifera L.). J. Gen. Virol. 91: 2524–2530.

De Miranda, J.R. & Fries, I. 2008. Venereal and vertical transmission of deformed wing virus in honeybees (Apis mellifera L.). J. Invertebr. Pathol. 98: 184–189.

Gauthier, L., Ravallec, M., Tournaire, M., Cousserans, F., Bergoin, M., Dainat, B., et al. 2011. Viruses associated with ovarian degeneration in Apis mellifera L. queens. PLoS One 6.

Highfield, A.C., El Nagar, A., Mackinder, L.C.M., Noël, L.M.-L.J., Hall, M.J., Martin, S.J., et al. 2009. Deformed wing virus implicated in overwintering honeybee colony losses. Appl. Environ. Microbiol. 75: 7212–7220.

Locke, B., Forsgren, E., Fries, I. & de Miranda, J.R. 2012. Acaricide treatment affects viral dynamics in Varroa destructor-infested honey bee colonies via both host physiology and mite control. Appl. Environ. Microbiol. 78: 227–235.

Moore, J., Jironkin, A., Chandler, D., Burroughs, N., Evans, D.J. & Ryabov, E. V. 2011. Recombinants between Deformed wing virus and Varroa destructor virus-1 may prevail in Varroa destructor-infested honeybee colonies. J. Gen. Virol. 92: 156–61.

Runckel, C., Flenniken, M.L., Engel, J.C., Ruby, J.G., Ganem, D., Andino, R., et al. 2011. Temporal analysis of the honey bee microbiome reveals four novel viruses and seasonal prevalence of known viruses, Nosema, and Crithidia. PLoS One 6: e20656.

Tentcheva, D., Gauthier, L., Zappulla, N., Dainat, B., Cousserans, F., Edouard, M., et al. 2004. Prevalence and Seasonal Variations of Six Bee Viruses in Apis mellifera L . and Varroa destructor Mite Populations in France. Appl. Environ. Microbiol. 70: 7185–7191.
