# Supplementary material for: Virus infections in honeybee colonies naturally surviving ectoparasitic mite vectors
Source: PLoS One. 2023 Dec 15;18(12):e0289883. doi: 10.1371/journal.pone.0289883 (PMC10723705; doi:10.1371/journal.pone.0289883)
Supplement: S1 File — (DOCX) [file pone.0289883.s003.docx]

**Virus infections in honeybee colonies naturally surviving ectoparasitic mite vectors**

**Sequences**

> DWV_So4_DWVF1425

GCTCCaCTGGAaTTCCTCAAGGTCGAGGTCGGTCAAGAAGCAGGCGAATGTATATTtaagAAGCCTAAATATACGCGCGTTTGCAAGAAAGTGAAGCGTGTTGCAACTCGCTTCGTTCGTGAAAAAGTTGTTCATCCTATGTGTTCTAGATCCCCTATGCTATTATTTAAGCTTAAGAAAATTATTTATGATTTGCACTTATATAGATTAAGAAAACAAATTAGAATGTTGAGACGTCAAAAACAGCGCGATTATGAGTTAGAGTGTGTCACTAATTTGTTACAATTATCGAATCCGGTGCAGGCAAAACCAGAGATGGATAACCCTAATCCAGGACCTGATGGCGAGGGTGAAGTTGAATTAGAAAAG

> BQCV_ Gr62_BQCVGFfwd

TACAATTCATATGATTTGGTTTCGAAGCGTTTTCCGTGGGCAAAAGTGGTTGGCTTAGTATACTCACCCCTGACAACAACTATTCCCGTAGATTTCATAGTATACGGTCATTTTGAGGACGTGGAGTTGGGTTGCCCAACTTCTGGGATGTTGGCTCAAGCCGGTCTGAAGGTGCAACCTCCTACAAACTCTGACTCTCGACAGCGTGCCAAAGAGAGTAAGGGCAAGAAGGATTACTCTAAGACAGGAGCAGCTTTTAAACAATCTGTTTCGAAGCTGGGCGAACATCTACCTTTCCTCAAATCAGGAGCGAACTGGGTCAGTGAGAATATTCTGCAACCTGCAGATGAAATCATAGGACCTATATTGTCTCTTTTTGGGTTCTCGAAGCCTCTTTTGCCCATTGCCAATCCTACCGTTTTACGGCCTGCAAATACCTTTGCTATCACTGATACCAACGATATGTCGCATAGTCTGGCGCT

>LSV-1_a6_qLSV1-F2569

CGATTCAACTCTCGTTACACTTGGCCCCTACTTTTCAGCTGTTGAGCATCGTGCTGCGTCCCA

>LSV-2_423_qLSV2-F1722

CGTTCCTCTTCCACACCCTTACCTGGTGACTCCTCTACGTCGGGATCAGTCTTACTACTCGACGACACTGCCGACCGAGTTCATGAGGATGACTCGTCCGCGAGATCGACACCGCA

>SBPV_3_F3177

AATCCAATATGGAATATTATGCGTGATGGTCATATTCCTGTTATTTCAAGTGGATTCAGGTATTTTAGAGGAGGATTACGACTCCGCATAGTTGTTGAAGGTCTTAATAGCTGTGTTTGGGTGCAACATCATCCTGATAGACCTAGTATATTTTCACGTCCTATAAT
