# Supplementary material for: Virus infections in honeybee colonies naturally surviving ectoparasitic mite vectors
Source: PLoS One. 2023 Dec 15;18(12):e0289883. doi: 10.1371/journal.pone.0289883 (PMC10723705; doi:10.1371/journal.pone.0289883)
Supplement: Attachment — Submitted filename: Plos one review_Rebuttal_2022_Lanz-Oddie.docx [file pone.0289883.s010.docx]

PONE-D-20-32132
Virus infections in Varroa destructor-resistant honeybees
PLOS ONE

This manuscript is being resubmitted after the decision to accept with major revisions. It was previously withdrawn to allow for the time needed to make the changes. Here we address the reviewer’s comments.

We have softened the claims, but do stand by our argument that the evidence presented does point toward a parasite resistance as the central mechanism for survivability.

Reviewer #1: This study reports analysis of prevalence of ten honey bee viruses (DWV, SBPV, BQCV, KBV, LSV1, LSV2, SBV, CBPV, IAPV and ABPV) in the colonies which selected for natural resistance to Varroa destructor, "Suppressed mite reproduction" (SMR), which survived without anti-Varroa treatments for 16 years, with those in with the colonies which received regular anti-varroa treatment until the year before the investigation. The analysis was carried out in Norway in 2013/2014 and involved 32 SMR (untreated) and 69 control (treated) colonies. This study showed that untreated (surviving) colonies had significantly lower levels of phoretic mite loads than susceptible colonies, which could explain lower prevalence of DWV-A in worker bees, considering that DWV is vectored by Varroa mites. Another interesting finding finding were higher prevalence ad titres of BQCV and LSV1 in the surviving colonies. The paper is well written and provides novel insight into mechanism of Varroa and virus tolerance, and the results are important for entomologists, invertebrate virologists and beekeeping community.

There are several questions which should be addressed:

lines 166-168.
Actual Varroa infestation data for phoretic mites and brood infestation data is required. An additional graph or table summarising Varroa loads informations should be included.

**Response:** A graph of the phoretic mite counts has been included. There was no significant difference between brood infestation rates, so this has not been graphed, but the statistical results added to the model table now included as Table 1.

Lines 128-132
Include sequences of the primers used for the virus detection and quantification as an additional Supporting Table.

**Response:** This has been added as a reference.

Lines 75-76/ Lines 128 -132
Provide full names of the viruses (DWV-A, DWV-B, SBPV, BQCV, KBV, LSV1, LSV2, SBV, CBPV, IAPV and ABPV)
when abbreviations are given first time in the text (Lines 75-76) rather than in lines 128 -132.

**Response:** This has been added.

Lines 204-208
Contradictory statements" "...No significant differences of virus prevalence were detected between susceptible and surviving pupae and mites for all tested viruses (Chi-Square: x2=0.76-3.17, p>0.05). Comparison of titers from susceptible and surviving pupae revealed that those from the susceptible colonies had significantly higher DWV-A titers..."
Does "all viruses" include DWV-A?

**Response:** The difference here is prevalence and titer. The statements are not contradictory; however a restructure of the statistical analyses has changed this result.

Line 39
"..DWV and its recombinants", it "recombinants" are mentioned this paragraph should mention that Therese are three types of DWV ( A, B. C) and there are recombinants between them. Probably easier will be to remove " and its recombinants".

**Response:** This has been addressed.

Reviewer #2: This manuscript presents a descriptive study comparing virus population profiles between selected mite-resistant honey bees and unselected mite-susceptible honey bees. Overall, the article addresses an important topic and is an incremental development of previous works about the association of parasitic Varroa mites and viruses in honey bee colonies. However, I have the following specific concerns with this study:
1. The hypothesis of the study is not clear or scientifically valid. The Varroa mite is unarguably the leading cause of western honeybee mortality worldwide through its direct damage inflicted on different developing stages of honey bees and its role as a vector of several viruses, in particular the Deformed wing virus (DWV). It has been well recognized that the prevalence, abundance and temporal changes of virus infections in honey bee colonies are influenced and highly correlated with the prevalence, abundance and length of Varroa infestation. While the change in virus landscape coincides with Varroa infestation, the evaluation of honey bees’ susceptibility and resistance should involve the measurement of host immune responses to viruses and virus replication dynamics in different groups of honey bees under controlled experimental conditions. The mere analysis of incidence, prevalence and abundance of viruses in two groups of honey bees is insufficient to draw any conclusions about the “tolerance” of honey bees to mite-vectored viruses. In my opinion, such conclusions are not supported by the data.
2. The name of subspecies in the present study needs to be specified.
3. There is a lack of information about the method used for absolute quantification of viruses (copy numbers). A standard curve is needed for each virus if an absolute quantification method was used.
4. A careful English editing throughout would considerably improve the manuscript.

**Response**

1. The paper has undergone a significant restructure, the statistical analyses have been completely redone and a table outlining the results of all models has been included in the main text. More care has been taken with the clarity of the entire document. Our central message is that the lower prevalence and titre of DWV-A in documented varroa-resistant colonies can be mostly explained by a lower mite load, but that tolerances to certain viruses, and susceptibilities to others cannot be excluded when describing the system.
2. If the reviewer refers to the subspecies of bee, it was a buckfast strain and the subspecies is not apparent, however we have specified that the domestic bees under observation were buckfast, likely crossed with carniolan.
3. This has been addressed.
4. English editing has been performed.
