# Supplementary material for: Virus infections in honeybee colonies naturally surviving ectoparasitic mite vectors
Source: PLoS One. 2023 Dec 15;18(12):e0289883. doi: 10.1371/journal.pone.0289883 (PMC10723705; doi:10.1371/journal.pone.0289883)
Supplement: Attachment — Submitted filename: Oddie etal2023_PlosOne_response to reviewers_MO4_yao_PN.docx [file pone.0289883.s011.docx]

Reviewer #1: The paper by Oddie et al. focuses on the analysis of viral infections (prevalence and viral loads) in Norwegian honey bee colonies that naturally survived infestation by Varroa destructor. They found lower V. destructor infestation levels in surviving colonies compared to Varroa susceptible colonies, and detected BQCV, DWV-A, LSV1, LSV2 and SBPV in all the colonies. The prevalence and viral load of Deformed Wing Virus (DWV-A) was lower in the mite surviving colonies in contrast to BQCV. Since the former virus is vectored by the mite they concluded that their findings suggests that lower mite levels may account for reduced prevalence and titers DWV-A rather than other mechanism of virus resistance in the surviving colonies

The study is interesting, well performed and described and it contributes to our knowledge on virus-resistance observed in V. destructor naturally surviving honey bee colonies. The conclusion is sound and it paves the way for further investigation of these mechanisms at the colony and individual bee levels.

The statement at lines 79-81 that indicates that the IKA viruses (IAPV, KBV and ABPV) and SBPV are not vectored by V. destructor should be changed since these viruses can be vectored by the mite as stated in Yañez et al. 2020, Bee Viruses: Routes of Infection in Hymenoptera.

Response: We firstly thank the reviewer very much for their time and effort. This correction has been made and the paper added to citations.

Reviewer #2: Editors,

The manuscript entitled, “Virus infections in honeybee colonies naturally surviving ectoparasitic mite vectors” by Oddie et. al describes differences in virus abundance between a reported samples obtained from colonies that are reportedly mite-resistant (i.e., survivor honey bee stock) compared to samples obtained from conventionally managed colonies. This is an interesting topic, but as currently presented it was too difficult to interpret the data to ensure accuracy, and the text requires additional work prior to publication.

Points to clarify or address before publication include:

1. Figures – In general, virus abundance data be better presented as box and whisker graphs that include and data point for all samples (i.e. rather than bar charts), so that readers can get a better understanding of the values and actual variability in the data.

Response: We thank the reviewer for thorough work and have endeavoured to address all points made:

We have added the points to the box and whisker plots as advised.

2. Abstract Line 21 / Introduction – While the citations in the introduction provide insight into mechanisms / behaviors associated with mite resistance and indicate that these adaptations can occur within 5 years (which seems short, if selection is acting at the colony level). It would be good for the authors to elaborate on this topic in regard to selection at the individual and/or colony level, etc. and/or revise the first line in their abstract and other place in the text. The researchers should be in other examples of hosts that have developed resistant to parasites over a similar relative time period (i.e., discuss typically ‘generation time’ for a honey bee colony, which is likely 1-2 years+ , and the number of years of mite exposure in Norway, etc.) in order to better describe how the mite-resistance described in this manuscript is/may be the result of natural selection.

Response: We thank the reviewer for highlighting these evolutionary aspects. There is consensus that European (-derived) populations of western honeybees, *Apis mellifera*, can survive infestations of *Varroa destructor* by means of natural selection This has been reviewed in detail by Locke 2016 and confirmed since, e.g. Oddie et al 2017 among many others. Clearly, selection at both individual bee and colony level are important in this regard as is the rather long generation time of honeybees. However, this has been dealt with in detail elsewhere (Locke 2016). So, here we just mention this briefly, cite the respective review accordingly, and prefer instead to focus on the possible role of the ability to cope with virus infections contributing to colony survival.

3. Abstract Line 22 - The Abstract should be changed since if the colonies were selected from mite resistance, that may result in lower viral load since mites are amplifying vectors of several honey bee viruses and likely passive vectors of many others – but that does not mean that the honey bees have developed resistance to the viruses. While the Abstract gets to this point it begins by stating “It is possible that resistance to the viruses spread by this parasite may contribute to colony survival,. . .” which is misleading/ confusing.

Response: We are afraid that the reviewer may have misinterpreted part of the matter: The surviving colonies investigated in this study were not selected for any mite resistance whatsoever. Instead, natural selection has enabled colony survival which may result from resistance / tolerance to mite infestations and our virus infections.

However, the reviewer is correct in another point: our main hypothesis is that viruses which are vectored by this mite should correlate with vector infestations levels and those not vectored by this mite should not. If the ability to cope with virus infections is an important factor contributing to colony survival, then virus levels should in general be low regardless of vector abundance and independent of a virus being vectored or not. On the other hand given that virus levels positively correlate with vector abundance and are equally high or even higher in non-mite-vectored viruses compared to mite-vectored ones, than the ability to generally cope with virus infections seems to be less relevant for colony survival than the ability to supress mite reproduction. Our data do indicate the latter. We have now rewritten the abstract to make this clearer to the readership.

The reviewer is also absolutely correct about the terminology. From our data, it cannot be concluded about resistance / tolerance whatsoever. Instead, we stick to "mite-surviving" as a well known colony phenotype regardless of the actual underlying mechanism.

4. Abstract – “The damaging variant of DWV, (DWV-A) .. “ is incorrect, both DWV-A and DWV-B can be detrimental to honey bees, as well as recombinant viruses.

Response: We have changed the terminology here to reflect the reviewer’s comment.

5. The authors need to include a list of primers used in this study, including the primers used to distinguish DWV-A vs. DWV-B. Were the viruses sequence? Were DWV-recombinants assessed? Without this information, some of the DWV abundance in this study may not be accounted for, and therefore the conclusions would only be based on some of the DWV. Regional differences in RNA viruses, which have required new primers for detection and quantification may be needed (e.g., see Daughenbaugh et al Viruses 2021 doi: 10.3390/v13020291, Moore et al. J. Gen. Virol. 2010 doi: 10.1099/vir.0.025965-0, and others).

Response: The paper now has a primer table in the supplementary files. More detailed information about the virus sequences were added to the text.

6. The Virus nomenclature needs to be revised throughout the manuscript, Lines 77-80+, to match ICTV guidelines. Virus nomenclature suggestion based on ICTV: , “black queen cell virus” and “deformed wing virus”, etc. be written in lower case, when referred to in-general (i.e., not a specific strain or species) should be written in lower case and not-italicized.
See: https://talk.ictvonline.org/information/w/faq/386/how-to-write-virus-species-and-other-taxa-names
Considering “deformed wing virus-A” is a virus name, “A virus name should never be italicized, even when it includes the name of a host species or genus, and should be written in lower case. The first letters of words in a virus name, including the first word, should only begin with a capital when these words are proper nouns (including host genus names but not virus genus names) or start a sentence.” Alternatively, for specific virus species, the authors could use “Deformed wing virus-A” (i.e., first letter capitalized, and italicized word), but this is rarely used.

Response: The virus nomenclature has now been carefully considered throughout the entire MS.

7. Figures, were all colonies assessed on the same date? The supplemental information is good, but would be improved by inclusion of sample date (not just season) or at least including the exact sample dates in the methods. Since the virus abundance in any colony changes over time.

Response: We thank the reviewer for highlighting this important point. Yes, indeed, seasonal fluctuations in virus levels are well-known. Therefore, we have of course taken this into account and sampled both mite-surviving and mite susceptible colonies in the same months. This has now been added.

8. The use of the word “titres” is inaccurate and should be revised throughout the manuscript.
Virus titer or viral titre = titre typically refers to the concentration of infectious viral particles
The authors present “viral abundance” or “viral load” data in the form of RNA or cDNA copies per bee (which would be better presented as copies/ xxx ng RNA), therefore the text should be revised.

Response: The entire text has been revised accordingly.

9. Virus abundance data is a major component of this study – yet it is unclear how this was determined in this study, and the reporting on figures, etc. requires major modification.

Specifically –
(a) Figure 3 – Log copies per colony is not the correct axis label.
It should be log(10) copies per 1000 ng total RNA (if they scaled results to represent total RT reaction).
The reaction volumes for RT and qPCR reactions should be included in the methods section and it should be clearly stated if Line 139 “3 ul cDNA” is really “3 ul of the 1:10 cDNA” – since, as written it is impossible to compare the virus copy number reported in this paper to other studies.
The researchers do not know the copy number per colony, since the assessment is done per x ul of a cDNA reaction – it represents some amount of RNA sample obtained from a subset of bees sampled from one colony.

(b) Lines 146 – 152, it is unclear how virus standards were made since simple dilutions of an unknown stock would not work to calculate “virus copy numbers” .
What did the authors use as a virus standard?
The standard data should be included and graphed to evaluate copy number and the qPCR efficiency.
PCR efficiency typically expressed as a percentage
(i.e., 10^(1/slope “3.32) – 1 = 1 or 100%
I am not sure how the authors calculated efficiency.

(c) The qPCR standard curves used to calculate virus abundance per qPCR well, and the math required to make that number representative of a given amount or RNA need to be clearly presented in this manuscript.

Response: We have addressed all of these concerns accordingly. In particular, the axis label on figure 3 has been changed to “Log copies per pooled sample”. More detailed information about the qPCR efficiencies and the standard curves were added to the text.

10. Virus abundance in individual colonies varies over time within a colony and the variation between individual colonies with the same apiary and/or region can be high.
The sample size in this study, which was n=10 mite-tolerant colonies, and n=11 conventionally managed colonies for virus abundance data was low. This sample sizes should clearly stated in the figures / figure legends The methods section reports higher numbers of both colonies in other sections making it difficult to see that the virus data came from a subset of the total number of colonies described.
The authors should revise the text describing putative virus resistance vs. tolerance since with a total of 21 colonies (i.e., 10 or 11 colonies per group) it would be difficult to try to assess the potential of virus resistance vs. tolerance.
For pupae and mite assessment it seems, 29 samples were obtained from mite resistant colonies located in three apiaries and 18 conventionally managed mite-susceptible colonies from five apiaries.
Since sample sizes vary for each figure, they must be included in the figures and figure legends, they should be included in every figure or figure legend.

Response: We agree with the sample size issue and are now much more careful in our data interpretation. Nevertheless, we are convinced that our data are solid. Again, we also agree with the virus resistance vs. tolerance issue (see above) and have removed this from the entire text. Finally, sample sizes have been added in the figure caption for each figure.

11. Discussion – Although the virus levels reported in honey bees in this study were lower in the “mite-resistant stock” were lower than the “conventionally managed susceptible stock” the number of mites were also lower – so the conclusion stated at the beginning of the discussion may not be accurate (i.e., Line 252), lower mite numbers may be associated with lower virus numbers.

Response: Again, we fully agree. The wording has been changed. Yes indeed, in our data low mite numbers correlate with low levels of the mite-vectored DWV-A thereby nicely confirming our hypothesis. We have now reworded accordingly.

12. As written the discussion is difficult to read, and included big overarching conclusions based on the data obtained from a relatively few number of colonies – that may or may not be exhibiting “mite-resistance” phenotype given that the levels of mite in the brood are similar in the different stocks (although they are missing the autumn time point).
There may be other areas to comment on in the discussion, but since the virus data was difficult to review/interpret it is not possible to fully evaluate the discussion.

Response: The mite survival of the colonies used for this experiment has been confirmed earlier and is referenced extensively in the introduction and discussion. In any case, we have substantially revised the discussion to make it clearer and more concise. In particular, we are much more careful now with our conclusions based on the sample size matter raised above.

Minor points to clarify or address before publication include:

1. Fig. 2 – y-axis does not need decimal places (e.g., 10%, instead of 10.00%)

Response: This has been addressed.
