# Supplementary material for: Virus infections in honeybee colonies naturally surviving ectoparasitic mite vectors
Source: PLoS One. 2023 Dec 15;18(12):e0289883. doi: 10.1371/journal.pone.0289883 (PMC10723705; doi:10.1371/journal.pone.0289883)
Supplement: Attachment — Submitted filename: Oddie-etal2023-Response to reviewer.docx [file pone.0289883.s012.docx]

Response to reviewer

Reviewer #2 comments:

The manuscript entitled, “Virus infections in honeybee colonies naturally surviving ectoparasitic mite vectors” by Oddie et. al was significantly improved by the review process.

Points to clarify or address before publication include:
1. While I understand that the abstract “European honeybee populations,
Apis mellifera, “ is indicating “Apis mellifera” populations in Europe, since some papers still define Apis mellifera as the European honey bee (as opposed to the Western honey bee) – the authors should revise the abstract to make sure this is clear – since PONE has a general readership.

Response:

The term in question meant to be very general and is meant to include all populations of *Apis mellifera*, as it is known that *A. m. scutellata* is very capable of persisting through *V. destructor* infestations. However, since this paper is wholly about a population actually bred in Europe, we have made this distinction.
