# Supplementary material for: Virus infections in honeybee colonies naturally surviving ectoparasitic mite vectors
Source: PLoS One. 2023 Dec 15;18(12):e0289883. doi: 10.1371/journal.pone.0289883 (PMC10723705; doi:10.1371/journal.pone.0289883)
Supplement: Attachment — Submitted filename: Oddie-etal2023-VirusVarroa_comments_2.2_clean.docx [file pone.0289883.s013.docx]

Reviewer #3: The paper by Oddie et al. analyzes viral infections (prevalence and viral loads) in Norwegian honey bee colonies that apparently survived infestation by Varroa destructor naturally. However, as a reviewer, I find the provided information insufficient to verify the authors' statement. The term "naturally survival" is not explicitly defined, and there is no information on the management system or procedures involved in the study. The lack of fundamental information, such as the size and strength of the colonies at the beginning of the experiment, feeding schedules, and honey collection, makes it difficult to evaluate the article's argument thoroughly.

The researchers found lower V. destructor infestation levels in surviving colonies compared to Varroa-susceptible colonies, which confirms the results from Dr. Oddie's previous work. The researchers detected BQCV, DWV-A, LSV1, LSV2, and SBPV in all the colonies. The prevalence and viral load of Deformed Wing Virus (DWV-A) was lower in the mite-surviving colonies, in contrast to BQCV. The authors concluded that their findings suggest that reduced mite levels may account for the lower prevalence and titers of DWV-A, rather than other mechanisms of virus resistance in the surviving colonies.

The study is interesting, but caution should be exercised when interpreting the results. In honey bee research conducted in the field, it can be challenging to obtain reliable control groups. Thus, it is uncertain what diseases or conditions were initially present in the colonies at the beginning of the experiment, which could interfere with the intended measurements.

Although the authors measured some pathogens, it is clear that unmeasured pathogens interfere with the replication of another pathogen, making it difficult to draw firm conclusions.

My suggestion is to include the missing information management information and perhaps perform an additional experiment by injecting bees with purified viruses and measuring replication efficiency in these two honey bee populations.

This experiment could provide the authors with a more robust indication of resistance selection mechanisms than those proposed in this manuscript.

Without more information, any conclusion regarding the mechanisms by which these "naturally" surviving honey bees survive varroa mite infestations is pure conjecture.

**Response**:

We thank the reviewer for the time and effort they placed in reviewing our paper.

Thew reviewer’s argument is well-founded, viral loads shift over seasons and time. We have softened the language around the findings and removed the hypothesis on viral tolerance focusing instead on the expected pattern in viral loads for parasite suppression.

Our observational studies were performed over the course of an entire year, so we do believe the most valuable shifts were captured in the data. We have provided further clarification as to the management and origins of the colonies, however the timing of management is defined by area, as temperature is dictated very much by the geography of the region and can separate seasonal patterns by as much as two weeks in as little a distance as 50km. The difficulty of standardizing management to collect meaningful data is an ever-present problem in field observations and should not be a deterrent to presenting observed patterns.

There are undoubtedly other pathogens affecting this system, however, the same can be said for all virus studies published, it is impossible to account for all interactions in a single study, and we feel this does not subtract from the legitimacy of the observational information presented in this paper.

The ability of this population to mitigate Varroa infestation levels is well-described in sourced papers and is not a subject of debate in this study.

We acknowledge that a manipulative experiment would support the findings but is not necessary in this case, as this is an observational study examining viral loads at the population level between two stocks with the fundamental difference of being adapted to *V. destructor* or not.

Reviewer #4: In this study, the authors hypothesized that in honey bee populations that survive V. destructor infestations without treatment, that is, that are "mite survivors," the bees may also have increased tolerance or resistance to viral infections vectored by the mite. The results presented here from analyzing honey bee colonies originating from the "mite-surviving" population and from a "normal", hence, mite susceptible population over three successive seasons in 2013/2014 are interesting, the paper is nicely written, however, some amendments and corrections are still necessary:

l. 16-17: The wording needs to be amended. This reads as if Western honey bees in general are known to survive V. destructor infestation. But this is not the case. It is true that there are reports and research on a few populations that survive mite infestation without treatment, e.g. in France, Sweden, Norway. But - to the best of my knowledge - these "adapted" bees lost their "adaptation" once transferred to other environments/regions of Europe.

**Response**: Firstly, we thank the reviewer for the time and effort they placed in reviewing our paper.

We have amended the abstract to clarify that we mean some populations and not all Western honeybees.

We are aware of a difference in experience between North American and European beekeeping in terms of *Varroa destructor* resistance, however the reviewer has received only a part of the picture presented by current literature:

The claim that adapted bees “lose” their adaptation is untrue and unrealistic in the context of evolution. The changes in environment and Varroa density provide challenges that can reduce the effectiveness of traits that have been adapted for a specific environment, therefore it has always been recommended to focus on breeding resistance in locally-adapted bees. We have restated this now in the paper (line 330-332).

There are many cases of untreated Western honeybees persisting in the face of Varroa, one of the latest published cases being the entire country of Cuba (Luis et al. 2022), where genetics studies revealed an almost exclusively European origin of the bees.

The argument of the loss of adaptation when bees are moved also falls drastically outside the scope of this paper, as there is no mention or encouragement of moving bees in any part of this paper.

l. 19-20: This is a very simplistic view on virus infections and host tolerance. Host induced reduction of viral titers may be one possibility but sustaining higher viral titers may be another one and selection of less virulent virus variants may be yet another one.
Please reword the sentence and make clear that you only test one of several different possibilities here.

**Response**: We have clarified that we mean to observe viral loads based on the known differences between these populations, we agree that this study is not sufficient to measure all possibilities of viral tolerance, we have also reworded the hypotheses to clarify our focus on the parasite suppression and its effect on viral loads.

l. 25-26: What about DWV-B? That is the relevant, clearly mite associated DWV-variant. Why are you concentrating on the less virulent variant DWV-A? Please explain and justify your choice of variant.

**Response**: DWV-B was discussed and tested for (line125), but when the data was collected it was not found in most of the samples. The reviewer must consider the spread of DWV-B and the timing of the study, coupled with the fact that Norway is a very closed country for honeybee imports and it is very possible this variant was not present in the population at the time.

l. 37: Please replace the term "recombinants" by the term "variants", because recombinants are only a subclass of the different variants within the DWV quasispecies.

**Response**: This has been amended.

l. 38: The wording is wrong: It is NOT the virus that developed new transmission routes but a new transmission route for DWV has opened up through the mite.

**Response**: This has been amended.

l. 45-47: The wording is misleading: Actually, small adapted populations have been reported from several European regions and have been analyzed for many years by now to identify the mechanisms behind these so far rather rare evolutionary events. Although these populations are worth to be studied, the sentence, as it reads right now, implies that every honey bee population would become "adapted" if left untreated. However, that such a development (evolution) would automatically occur within 5 years, if the colonies would be left untreated, has never been shown. Moreover, bee colonies from these allegedly mite-tolerant populations were no longer able to survive mite infestation without treatment when brought to other regions in Europe. The question still remains whether it is the bee or the mite or both that adapt to "lack of treatment".
Therefore, I request that the authors rephrase this sentence in a way that it better reflects the current knowledge and does not leave the impression that stopping mite treatment for five years will solve the mite problem and result in mite surviving honey bees all over Europe.

**Response**: We disagree with the reviewer’s claims here. There is a very abundant body of literature that provides evidence for the ability of Western honeybees to adapt to Varroa and we have cited it. We acknowledge the fundamental differences in experience between North American and European beekeeping operations, but this should not be a factor that dictates the interpretation of previous peer-reviewed evidence. Our wording was cautious, and “would develop” was not used, but “can” indicating the possibility but not a definite outcome. It has been observed to occur in as little as 5 years, this is in the literature cited. To the argument that honeybees “lose their adaptation when moved”, we refer to the argument and cited papers above.

We believe the language is sufficiently cautious given the evidence we have presented, there are no claims that stopping mite treatments will result in resistant bees indefinitely, and we will not be changing the wording here.

l. 54-55: Especially the Primorsky bees and the Gotland bees were tested for survival without Varroa treatment in Germany and they were no longer able to survive mite infestation without treatment. This has been published and should not be ignored because it is a very interesting aspect regarding the evolution of mite tolerance in honey bee populations.

**Response**: Here the reviewer likely refers to a paper written in 2004 by Berg et al. referenced below, however there was another study published on this same stock moved to the United States which showed a retention of the trait and the subsequent creation of the Russian Honeybee Breeder’s Association in the US (Rinderer et al. 2010), also referenced below.

We have cited both these papers and insist that Varroa resistance is a practical and realistic strategy as long as biology and local environments are considered.

Berg S., Fuchs S., Koeniger N., Rinderer T.E. (2004) Preliminary results on the comparison of Primorski honey bees, Apidologie 35, 552–554.

Rinderer TE, Harris JW, Hunt GJ, De Guzman LI: (2010) Breeding for resistance to Varroa destructor in North America. Apidologie 41:409-424 <http://dx.doi.org/10.1051/apido/2010015>.

We restate that the argument of moving bees does not apply to this paper, resistant bees have been shown to keep their resistance consistently when managed in the areas where they were bred. This has been documented in scientific literature in at least 8 independent cases across the globe.

l. 78: SBV and CBPV are known to be vectored by V. destructor, please read the literature.
That the vector capacity of the mite has not yet been shown for the other viruses mentioned does not mean that the mite is not able to vector them. There are just too few comprehensive studies on viruses in mites. Please correct and rephrase.

**Response**: The reviewer cannot claim that these viruses are vectored by Varroa, only associations have been shown in previous literature and these associations are not consistent. According to a paper published in 2022 (O’Shea-Wheller) CBPV can be associated with Varroa but viral transmission pathways have been “elusive”, citing two others: Seitz et al. (2019) and Ribière et al (2010), the same associations were found for SBV (Shen et al. 2005). We have edited the text to describe this association. If the reviewer insists on both viruses being actively vectored by Varroa, we ask that they provide literature where this has been shown, otherwise we must adhere to the consensus provided by the scientific literature found.

l. 172: It would be better to use the same terminology in the text and in the table, i.e. either worker infestation rate or phoretic mite infestation levels. Please correct.

**Response**: This has been amended.

l. 174: Will the supporting information (raw data in VirusVarroa.zip) also be available to the reader? If so, please refer here to this information but remove the names of the beekeepers in the file.

**Response**: All data is uploaded and available as per the journal’s requirements. The names of the beekeepers and locations have been changed to provide anonymity.

l. 182: (i) Please indicate whether mean +/- SD or +/- SEM is shown. (ii) The number of the colonies differs considerably between spring, summer, autumn. Why? Did colonies die? Did all "surviving" colonies really survive the winter season and how many of the "susceptible" colonies did not survive the winter season? (iii) Acc to MatMeth, the samples were collected in Sept 2013, April 2014 and June 2014. I assume that spring is April 2014, summer is June 2014 and autumn is Sept 2013. The proper order of the seasons in the figure then were autumn, spring, summer. Please rearrange and add the year. It will then become clear that information on winter mortality must exist because the colonies were observed over winter. Please provide this information. It is absolutely necessary for interpreting the data. Are the “surviving” colonies really surviving, are the susceptible colonies really more susceptible (more of them collapsing over winter) than the “surviving” colonies? Plesase add the requested information and consider it in the interpretation of your data.

**Response**: We have now indicated that the standard errors are shown.

The number of colonies differ due to reasons including loss, accessibility and beekeeper permissions, some colonies are moved onto heather in the local migratory practices. Winter loss data was not collected and falls outside the scope of the paper, because, as stated above, the fact that these colonies can survive mite infestations has already been published in peer-reviewed work multiple times and is not a subject for debate, nor an argument made by the data presented, it is taken as a well-studied fact. Please refer to the numerous studies cited in this work.

l. 194: I assume that all colonies that were sampled in April and June 2014 survived the winter 2013/2014. Hence, strictly speaking, all colonies were "surviving" colonies". If this is not the case, please specify and differentiate between truely surviving colonies and replaced colonies in both groups.
I think it is better to keep writing mite-surviving and mite-susceptible colonies because otherwise the impression is given that the other colonies have not survived - but they had, because they were sampled!? Or didn't they? Then please specify.
Please amend the terminology throughout the manuscript.

**Response**: Please refer to above comment, this has been addressed.

l. 202, test stat (X2) 15.30: In Table 1, the value 15.03 is given. Please correct. Please explain "(from qpcr negative results)" given in the Table.

**Response**: This has been amended. Prevalence taken from the absence or presence of virus in the qpcr analysis, binary presence absence tests were not performed, only the quantitative pcrs but this is sufficient to show the presence or absence of a virus.

l. 203, test stat (X2) 6.75: In Table 1, the value 4.61 is given. Please correct.

**Response**: This has been amended.

l. 203, p value 0.009: In Table 1, the value 0,03 is given. Please correct.

**Response**: This has been amended.

l. 204: Acc to MatMeth, for virus prevalence pooled samples were collected in April and June 2014. Please provide this information somewhere in the legend of the Fig and explain/justify why you combined the results from two sampling time points.

**Response**: This has been amended. The graph depicts prevalence for viruses sampled in June which are most relevant. As season did not affect most viruses, this was decided as the most concise way of conveying the important results. We have clarified the season in the figure caption.

l. 209, figure 4: It must be Fig. 3. Please correct.

**Response**: This has been amended.

l. 217: Please stick to one terminology! For instance, either write “mite-surviving colonies and mite-susceptible colonies” or “colonies from the mite-surviving population and the mite-susceptible population”, but don't switch between susceptible, mite-susceptible, treated etc. colonies.
This is especially relevant if you use a term here (treated) that cannot be found in the Figure or elsewhere.

**Response**: Changes have been made to reinforce “surviving” and “susceptible” as the central terminology, however, sometimes these terms are accompanied with “untreated” and “treated” to emphasize the fact that the colonies that have resistance to the parasites are not given any treatment for varroa. This is an important distinction and we press that it should remain.

l. 223: I cannot find results for individual adult workers in Table 1, only for (i) individual worker brood, (ii) pooled adult worker, and (iii) individual viral loads pupae: Likewise searching for X2 33.751 did not yield a result. Please correct.

**Response**: These data were analysed using a χ^2^ test and not a glm. Therefore table 1 should not have been referenced. The text has been amended in methods and results.

l. 225: Please be consistent: If the legend of the axis says "wandering mites" don't use "phoretic mites" in the figure legend.

**Response**: There is no mention of “wandering” mites in text or in figures or tables. Phoretic is used for mites on adult worker bees and the term “associated” is used for mites found in brood to indicate that the mite and brood samples were paired.

l. 238: For pupae, I can only find data for individual viral loads but not for prevalence in Table 1. Please correct.

**Response**: Models 3a-c display the data for individual pupae and mite sample prevalence. These models have been set lower in the table to reflect the order of presentation in text and are now models 5a-c.

l. 240: In Table 1 the term "viral load" is used, here it is "virus abundance". Please be consistent and rather take viral load throughout.

**Response**: Previous reviews had the term changed to “abundance”, we have now made this consistent throughout the manuscript and supporting documentation.

l. 246: Fig. 5 is about prevalence and abundance, not only prevalence. “Abundance” is better named viral load. Please correct.

**Response**: Figure 5 is strictly about abundance, the text has been corrected, please see above comment in regards to chosen terminology.

l. 246: Fig. 5 is supposed to show surviving pupae and associated mites (see l. 241), not adult workers and phoretic mites. Please correct.

**Response**: This has been amended.

l. 247: Fig 5 is a box plot showing more than just means. Please describe correctly what is shown and specify the box and error bars (SD? SEM?).

**Response**: This has been amended.

l. 249: Here the use of "surviving" colonies is especially problematic. Since these data are from autumn, the use of the term surviving implies that the data of surviving colonies from both groups (mite survivjng and mite susceptible) are shown here, but I assume that surviving actually means “mite surviving”. Please correct.

**Response**: This has been addressed in previous comments: Winter loss data was not collected and falls outside the scope of the paper, because, as stated above, the fact that these colonies can survive mite infestations has already been published in peer-reviewed work multiple times and is not a subject for debate, nor an argument made by the data presented.

l. 256: Please provide data on what happened over winter. Did the described differences had any influence on the mortality rate? Did the highly infested and virus infected colonies all die or did they survive? This information is lacking but highly relevant. Please add this information.

**Response**: This has been addressed in previous comments.

l. 259: Please also mention and properly discuss the possibility that the experimental design (pools of entire bees were analyzed, only DWV-A was analyzed instead of DWV-B or even better both variants) was not optimal for answering the question.

**Response**: This has been addressed in previous comments.
