# Supplementary material for: Virus infections in honeybee colonies naturally surviving ectoparasitic mite vectors
Source: PLoS One. 2023 Dec 15;18(12):e0289883. doi: 10.1371/journal.pone.0289883 (PMC10723705; doi:10.1371/journal.pone.0289883)
Supplement: Attachment — Submitted filename: Oddie-etal2023-VirusVarroa_comments_2.3_clean.docx [file pone.0289883.s014.docx]

Journal Requirements:

Please review your reference list to ensure that it is complete and correct. If you have cited papers that have been retracted, please include the rationale for doing so in the manuscript text, or remove these references and replace them with relevant current references. Any changes to the reference list should be mentioned in the rebuttal letter that accompanies your revised manuscript. If you need to cite a retracted article, indicate the article’s retracted status in the References list and also include a citation and full reference for the retraction notice.

**Author comment:** To our knowledge, none of the references cited have been retracted, species names have been italicized as per the editor’s request.

Additional Editor Comments:

I can see that this ms has been reviewed by 4 people at various times and 3 suggest minor revisions. The final and latest one suggested major revision which I am satisfied you have addressed fully in your changes to the final versions and in the response letter. As the new editor for the ms I have carefully read the most recent version and suggest the following minor changes before final acceptance as I am not requesting any more reviews so these are the final changes.

Response: We are very happy with the editor reassignment and thank you very graciously for the feedback.

Line 39 remove full stop after ‘many cases’
Line 81 reword (BQCV and LSV),
Line 104 Change 100 to ‘One hundred’
Line 272, maybe add 'as suggested by Grindrod and Martin (2021)'?.
Line 327 remove full stop before refs
Check refs for Latin names and put in italics.

Both these previous studies found lower DWV viral loads in resistant populations so worth adding into your discussion as it strengthens your findings:
de Souza FS, Allsopp M, Martin SJ (2020) Deformed wing virus prevalence and load in honeybees in South Africa. Arch Virol (166): 237–241.

Mendoza Y et al., (2020) Unravelling Honey Bee–Varroa destructor Interaction: Multiple Factors Involved in Differential Resistance between Two Uruguayan Populations. Vet Sci (7):116.

Also, Grindrod and Martin (2021) states in the discussion that “a reduced mite burden also reduces the number of viral vectors” and you come to the same conclusion so maybe worth citing this ref in that context to support your ideas.
Grindrod I, Martin SJ (2021) Parallel Evolution of Varroa Resistance in Honey Bees; a common mechanism across continents? Proc. R. Soc. B 288: 20211375.

**Response:** All changes have been made precisely as the editor has specified, new references have been cited and discussed in text and all minor edits have been made (line numbers match those specified)
